# Influence of weight gain, according to Institute of Medicine 2009 recommendation, on spontaneous preterm delivery in twin pregnancies

**DOI:** 10.1186/s12884-017-1645-5

**Published:** 2018-01-03

**Authors:** Paola Algeri, Francesca Pelizzoni, Davide Paolo Bernasconi, Francesca Russo, Maddalena Incerti, Sabrina Cozzolino, Salvatore Andrea Mastrolia, Patrizia Vergani

**Affiliations:** 1 Department of Obstetrics and Gynecology, University of Milano-Bicocca, S. Gerardo Hospital, MBBM Foundation, Via Pergolesi 33, Monza, 20900 Monza, Monza e Brianza Italy; 2 Department of Health Sciences, Center of Biostatistic for Clinical Epidemiology, University of Milan-Bicocca, Via Pergolesi 33, Monza, 20900 Monza, Monza e Brianza Italy

**Keywords:** Twin pregnancy, Preterm delivery, Preterm labor, Weight gain, Institute of medicine recommendation

## Abstract

**Backgrounds:**

Maternal total weight gain during pregnancy influences adverse obstetric outcomes in singleton pregnancies. However, its impact in twin gestation is less understood. Our objective was to estimate the influence of total maternal weight gain on preterm delivery in twin pregnancies.

**Methods:**

We conducted a retrospective cohort study including diamniotic twin pregnancies with spontaneous labor delivered at 28 + 0 weeks or later. We analyzed the influence of total weight gain according to Institute of Medicine (IOM) cut-offs on the development of preterm delivery (both less than 34 and 37 weeks). Outcome were compared between under and normal weight gain and between over and normal weight gain separately using Fisher’s exact test with Holm-Bonferroni correction.

**Results:**

One hundred seventy five women were included in the study and divided into three groups: under (52.0%), normal (41.7%) and overweight gain (6.3%). Normal weight gain was associated with a reduction in the rate of preterm delivery compared to under and over weight gain [less than 34 weeks: under vs. normal OR 4.97 (1.76–14.02), over vs. normal OR 4.53 (0.89–23.08); less than 37 weeks: OR 3.16 (1.66–6.04) and 6.51 (1.30–32.49), respectively].

**Conclusions:**

Normal weight gain reduces spontaneous preterm delivery compared to over and underweight gain.

## Background

Pre-gestational body mass index (pBMI), gestational body mass index (gBMI) and total weight gain influence the incidence of preterm delivery and other adverse obstetric outcomes in singletons [1, 2] but their effect on twins is poorly understood, although multiple pregnancies appear to have similar associations between these outcomes, pBMI or weight gain compared to singletons [1, 2]. Indeed, studies evaluating the role of both total and weekly weight gain in twin pregnancies identified a strong correlation between low total weight gain during pregnancy and preterm delivery (PD) [3, 4]. In 1990, the Institute of Medicine (IOM) proposed ranges of recommended total weight gain correlated to pBMI for singleton pregnancy and an optimal total weight gain between 15.9 and 20.5 kg not related to pBMI for twin pregnancy [5, 6].

In 2009, the IOM revised these guidelines and defined pBMI specific weight gain cut-off, also for twin pregnancies. Optimal ranges proposed for weight gain at term (≥ 37 weeks) are: 17–25 kg for normal weighted women (pBMI 18.5–24.9), 14–23 kg for over weighted women (pBMI 25–29.9) and 11–19 kg for obese women (pBMI 30 or more). No recommendations were given for underweighted women (pBMI less than 18.5) [7].

This is of higher importance due to the rise in the incidence of twin pregnancies in the last three decades because of the older age at childbearing and of the diffusion of assisted reproductive technology [8]. Today, approximately 1 of 80 pregnancies is a multiple gestation, corresponding to 2.6% of all newborns (1–3% in Italy) and they are more frequently diamniotic. Multiple pregnancies present a higher incidence of maternal and fetal adverse outcomes compared to singleton ones [3, 9–13]. Twin pregnancies account for 12.2% of preterm births and 15.4% of neonatal deaths [14–16].

In literature, both pBMI and total weight gain were reported as important influencing factors in pregnancies outcomes. However, the studies took into consideration only one of these parameters at a time. New cut-offs proposed by IOM allowed an easier evaluation of maternal weight influence on obstetrics outcomes, not only in singleton pregnancies but also in twins.

Few studies evaluated the role of the new IOM guidelines in influencing preterm delivery in twins, also considering that IOM gave cut-offs only for gestational age at delivery ≥ 37 weeks [17–19].

In 2010, Fox et al. conducted a study on a cohort of twins divided into subgroups considering pBMI. They show that patients whose weight gain during pregnancy met or exceeded the revised 2009 IOM guidelines had significantly improved pregnancy outcomes such as longer gestation, less overall PD, less spontaneous PD and larger neonates compared to lower weight gain [18]. In 2012, also Quintero et al. found that a weight gain below recommended guidelines was associated with higher rates of spontaneous PD at less than 35 weeks in twin pregnancies [19].

A recent review tried to define the role of absolute total weight gain in the development of adverse pregnancy outcomes. The authors suggested that a higher incidence of PD was correlated with underweight gain and underlined a positive correlation between total weight gain and gestation length [20].

Gestational gain weight and pBMI have been proven to influence not only the risk of PD but also other obstetric outcomes, such as birth weight, hypertensive disorders, gestational diabetes and neonatal adverse outcomes [17].

Contrasting results were instead reported about hypertensive disorders: while some authors described higher incidence of gestational hypertension and preeclampsia in women with excessive weight gain, others showed no differences among different weight gain groups in a series of twin pregnancies delivering at term [5, 17, 20].

In light of the above and due to the scarcity of data available in the literature about this topic, we designed a study with the aim to estimate the influence of total weight gain according to the 2009 IOM recommendations on preterm delivery before 37 and 34 weeks in twin pregnancies with spontaneous onset of labor. Secondary outcomes were the possible correlation with small for gestational age (SGA) and large for gestational age (LGA), pregnancy hypertensive disorders, gestational diabetes, and neonatal adverse outcomes.

## Methods

We performed a retrospective cohort study on diamniotic twin pregnancies delivered at more or equal 28 + 0 weeks after spontaneous onset of labor at our Institution (Fondazione MBBM, San Gerardo Hospital, University of Milano Bicocca, Monza, Italy), between January 2010 and December 2013.

Exclusion criteria of our study were induction of labor (15% of all twin pregnancies at our Institution), elective cesarean section (4% of all twin pregnancies at our Institution), monoamniotic twins, intrauterine demise, fetal malformations, twin-to-twin transfusion syndrome, and gestational age at delivery < 28 weeks. We decided to set a gestational age < 28 weeks at delivery as an exclusion criteria in order to have a better definition of the weight gain trend for each patient. A shorter pregnancy duration could be a confounding factor in defining the maternal weight gain.

Patients with pBMI < 18.5 were also excluded since there are no IOM recommendations for underweight patients in case of multiple pregnancies.

All twin gestations were followed according to national guidelines for management of twin pregnancy [21]. The protocol included maternal clinical assessment and ultrasound monitoring every 2 weeks, from 16 weeks, for monochorionic diamniotic pregnancies and every 4 weeks, starting from 20 weeks, for dichorionic diamniotic gestations.

At our Maternal-Fetal Unit, women undergo their first access at obstetric booking that is usually performed during the first trimester after a positive pregnancy test (<8 weeks of gestation). At the first visit we collect patient’s medical and obstetric history, define gestational age (calculated based on the last menstrual period and confirmed by ultrasound assessment), as well as chorionicity. Baseline characteristics and pregnancy outcomes were entered into our database by an assigned physician at every patient’s access and periodically reviewed by a senior consultant. pBMI was recorded at the first visit, and maternal weight was measured at each obstetric control until delivery.

Since self-report of pBMI can be affected by recall bias, we attempted to reduce the risk of bias with an early assessment of pregnant women as described above.

Total weight gain was calculated as the difference between maternal weight at delivery and pre-gestational weight. This parameter was used to classify women who delivered at 37 weeks or more according to IOM guidelines [7]. In case of preterm delivery (between 28 and 36 + 6 weeks), we calculated a weekly weight gain cut-off as total weight gain during pregnancy in kg/gestational weeks at delivery. We compared this weekly weight gain to a hypothesized weekly IOM cut-off, calculated as IOM cut-off at term/37 weeks, as previously reported, represented for normal-weight women, this was 1.0 lb. per week (37 lbs. over 37 weeks); for overweight women, this was 0.84 lb. per week (31 lbs. over 37 weeks); for obese women, this was 0.68 lb. per week (25 lbs. over 37 weeks) [18].

The data used for the analysis were already available for every patient as part of the clinical report of the Obstetric Department.

SGA and LGA were defined, respectively, as neonatal weight at birth < 10° centile and > 90° centile compared to Italian Neonatal Study (INeS) charts [22]. We considered as separate outcomes the occurrence of at least one twin SGA/LGA and both twins SGA/LGA.

We defined “gestational hypertensive disorders” as the presence of at least one among gestational hypertension, preeclampsia or eclampsia, diagnosed according to American Congress of Obstetricians and Gynecologists (ACOG) criteria [23].

Gestational diabetes was defined as any degree of glucose intolerance with onset or first recognition during pregnancy [24].

We defined composite adverse neonatal outcome as the presence of at least one among: need for neonatal resuscitation, respiratory distress syndrome, disseminated intravascular coagulation, intra-ventricular hemorrhage, leucomalacia, sepsis, necrotizing enteritis, retinopathy of prematurity and neonatal death.

The present work was exempt from IRB approval as per Institutional policy on retrospective studies. At our medical center, women provide a written consent to the use of their clinical anonymized and de-identified data upon admission.

### Statistical analysis

Population characteristics were compared among IOM weight gain groups using Chi Square test (categorical variables) or One Way ANOVA (continuous variables). Primary and secondary outcomes rates were compared between under and normal gain and between over and normal gain separately using Fisher’s exact test with Holm-Bonferroni correction. Logistic regression analysis was carried out in order to evaluate the independent effect of weight gain adjusted for pBMI on the outcomes. A separate model was built for each primary and secondary outcome. All the analyses were performed using the R software, version 3.0.2. A *p* value of less than 0.05 was considered significant.

## Results

The incidence of twin pregnancies at our Institution was 2.5–3% during the study period.

A cohort of 175 diamniotic twin pregnancies was included in our study, considering exclusion criteria: 91 (52.0%) presented underweight gain, 73 (41.7%) normal weight gain and 11 (6.3%) over weight gain, according to IOM recommendations.

Table 1 shows general population characteristics in the three study groups, considering the IOM classification for total weight gain. The normal weight gain group had a higher mean gestational age at delivery compared with the under and over gain weight ones (respectively 36.5 ± 2.0, 35.3 ± 3.0, 35.3 ± 2.0 weeks). Normal and overweight gain patients presented higher neonatal weight at birth for both twins compared to the under gain ones (respectively 2494.11, 2974.55, 2196.54 g). The over weight gain group presented a higher incidence of pre–gestational over weighted patients (45.5%). The study groups did not differ for other characteristics.

**Table 1:** Population general characteristics, according to weight gain groups

	Under (91)	Normal (73)	Over (11)	*P* value
Maternal age	34 ± 5.55	34 ± 5.86	34 ± 5.03	0.99
Nulliparity	52 (57.1%)	44 (60.3%)	8 (72.7%)	0.32
Smoker	3 (3.9%)	5 (6.8%)	1 (9.1%)	0.38
Chronic Hypertension	1 (1.1%)	1 (1.4%)	0	0.93
pBMI^a^	22.75 ± 4.44	23.00 ± 3.29	23.97 ± 2.50	0.70
18.5 ≤ pBMI^a^ ≤ 24.9	74 (81.3%)	54 (74.0%)	6 (54.5%)	0.11
25 ≤ pBMI^a^ ≤ 29.9	10 (11.0%)	15 (20.5%)	5 (45.5%)	*0.01*
pBMI^a^ ≥ 30	7 (7.7%)	4 (5.5%)	0	0.57
Medically assisted procreation	16 (17.6%)	18 (24.7%)	4 (36.4%)	0.26
Mono- Chorionicity	13 (14.3%)	11 (15.1%)	2 (18.2%)	0.94
Clinical chorionamnionitis	1 (1.1%)	0	0	0.63
Preterm rupture of membranes^b^	30 (33.0%)	13 (17.8%)	3 (27.3%)	0.09
Gestational age at delivery (weeks)	35.3 ± 3.0	36.5 ± 2.0	35.3 ± 2.0	*0.002*
Vaginal delivery	34 (37.4%)	23 (31.5%)	4 (36.4%)	0.73
1^st^ twin birth weight (gr)	2196.54 ± 592.16	2494.11 ± 426.87	2374.55 ± 471.63	*0.002*
2^nd^ twin birth weight (gr)	2154.89 ± 499.41	2392.95 ± 413.94	2443.50 ± 334.47	*0.002*

Results are reported as means and standard deviations (continuous factors) or numbers and percentages (categorical factors). The *p*-value of an overall test comparing the three groups is also provided (One-way ANOVA for continuous factors and Chi-square test for categorical factors)

Italic data are statistically significant

^a^pBMI = pre-gestational Body Mass Index; ^b^Preterm rupture of membrane = rupture before 37 weeks

The incidence of primary and secondary adverse outcomes was compared among the three groups, and the results are presented in Tables 2 and 3.

**Table 2:** Incidence of the primary outcomes in the weight gain groups

	Under (n. 91)	Normal (n. 73)	*p*-value§	Over (n. 11)	Normal (n. 73)	*p*-value§
Preterm delivery < 37 weeks	61 (67.0%)	29 (39.7%)	*P = .002*	9 (81.8%)	29 (39.7%)	*P = .04*
Early preterm delivery < 34 weeks	23 (25.3%)	5 (6.8%)	*P = .005*	3 (27.3%)	5 (6.8%)	*P* = .13

Results are reported as numbers and percentages. The *p*-value of an overall test (Chi-square test) correlating the three groups for the two by two comparison

Italic data are statistically significant

§Fisher’ exact test with Holm-Bonferroni correction

**Table 3:** Incidence of the secondary outcomes in the weight gain groups

	Under (n. 91)	Normal (n. 73)	Over (n. 11)	*P* value^c^
At least one twin SGA^a^	16 (17.6%)	13 (17.8%)	0 (0%)	0.31
Both twins SGA^a^	4 (4.4%)	1 (1.4%)	0 (0%)	0.43
Hypertensive disorders	7 (7.7%)	13 (17.8%)	7 (63.6%)	*< 0.001*
Gestational diabetes	15 (16.5%)	7 (9.6%)	0	0.18
1st twin adverse outcomes^b^	20 (22.0%)	6 (8.2%)	2 (18.2%)	0.06
2nd twin adverse outcomes^b^	20 (22.0%)	8 (11.0%)	2 (18.2%)	0.18

Results are reported as numbers and percentages

Italic data are statistically significant

^a^SGA = small for gestational age; ^b^Adverse outcomes = neonatal resuscitation, respiratory distress syndrome, disseminated intravascular coagulation, intra-ventricular hemorrhage, leucomalacia, sepsis, necrotic enteritis, retinopathy of prematurity; ^c^Fisher’s exact test with Holm-Bonferroni correction, for all comparison; ns, not significant

We found that the normal weight gain group presented a significant lower incidence of spontaneous PD compared to both under and over weight gain groups [respectively 39.7% vs 67.0% (p: 0.002); 39.7% vs 81.8% (p: 0.04)]. Underweight gain women presented significantly higher rates of early preterm spontaneous delivery compared to normal weight gain ones; a trend was also reported when overweight gain was compared to normal weight gain group [respectively 25.3% vs. 6.8% (p: 0.005); 27.3% vs. 6.8% (p: 0.13)].

No differences in the occurrence of SGA in one or both twins were observed among the three study groups. In addition, no cases of LGA were recorded. Gestational hypertensive disorders occurred in our population included 1 case of gestational hypertension and seven cases of preeclampsia in the underweight group, five and eight respectively in the normal weight, and one and six in the overweight one. No cases of eclampsia were reported. In the normal weight gain group, women presented a trend toward a higher incidence of hypertensive gestational diseases compared to underweight gain patients, even if not significant (17.8% vs. 7.7%, *p* = 0.06). This complication was significantly less frequent in the normal weight gain group compared with the over weight gain one (17.8% vs. 63.6%, *p* = 0.006). No difference in the incidence of gestational diabetes mellitus and neonatal adverse composite outcomes were reported in the three study groups, and we had no neonatal deaths.

The results of the bivariate analysis on both primary and secondary outcomes were confirmed in the multivariate logistic regression analysis, adjusting for the effect of pBMI (Tables 4 and 5, respectively). Both under and over weight gain increased the risk of PD compared to normal weight gain: ORs were 4.97 (1.76–14.02) and 4.53 (0.89–23.08) for early preterm and 3.16 (1.66–6.04) and 6.51 (1.30–32.49) for PD at less than 37 weeks (Table 4). Women in the underweight gain group had a lower risk of developing hypertensive gestational disorders compared to the women of the normal weight gain group, even if it was not significant, but only a trend (OR = 0.39, 0.15–1.05). The over weight gain group, instead, presented a significantly higher risk of hypertensive gestational disorders compared to the normal weight gain group (OR = 7.69, 1.94–30.47).

**Table 4:** Effect of weight gain on the primary outcomes estimated by logistic regression

	Early preterm delivery OR (95%CI)	Preterm delivery < 37 weeks OR (95%CI)
Under vs normal	4.97 (1.76; 14.02)	3.16 (1.66; 6.04)
Over vs normal	4.53 (0.89; 23.08)	6.51 (1.30; 32.49)

The models were adjusted for pre-pregnancy BMI

**Table 5:** Effect of weight gain on the secondary outcomes estimated by logistic regression

	At least one twin SGA^a^ OR(95%CI)	Hypertensive gestational disorders OR(95%CI)	Gestational diabetes mellitus OR(95%CI)	Neonatal adverse outcomes^b^ OR(95%CI)
Under vs. normal	1.01 (0.45; 2.28)	0.39 (0.15; 1.05)	1.98 (0.75; 5.22)	0.94 (0.29; 3.05)
Over vs. normal	–	7.69 (1.94; 30.47)	–	0.44 (0.05; 3.82)

The model for “neonatal adverse outcomes” was adjusted for pre-pregnancy BMI and gestational age. All the other models were adjusted only for pre-pregnancy BMI

^a^SGA = small for gestational age; ^b^Adverse outcomes = neonatal resuscitation, respiratory distress syndrome, disseminated intravascular coagulation, intra-ventricular hemorrhage, leucomalacia, sepsis, necrotic enteritis, retinopathy of prematurity

## Discussion

### Principal findings of the study

In this study, we evaluated the influence of total maternal weight gain, according to the revised IOM recommendations, on the development of spontaneous PD in diamniotic twin gestations [7]. Our results show that 1) normal weight gain is associated with a significant reduction in preterm parturition; and 2) when taking into consideration gestational age at delivery, both under and overweight gain groups presented an increased risk of early preterm parturition compared to normal weight gain women; and 3) a significantly increased risk for preterm parturition before 37 weeks (three and six times respectively) was present in underweight and overweight women respectively, compared with normal weight gain women.

#### IOM recommendations for weight gain in twin pregnancies

The important novelty of 2009 IOM recommendations was to give pBMI correlated cut-offs in twin pregnancies. On the other side, a limitation on the clinical use of these guidelines was to refer only to term twin pregnancies, excluding a twin group that delivered before 37 weeks [7, 17]. Therefore, just because IOM guidelines may be used limitedly to term twin pregnancies, we wanted to value how to apply them also in preterm gestations. Thus, we used a weekly gain weight cut-off (IOM cut-off at term/37 weeks), as already done by Fox et al. in a previous study [18].

#### Available literature assessing the influence of weight gain on pregnancy outcomes, in twin gestations

Several studies [17–19] analyzed the influence of IOM recommendations on pregnancy outcomes in twin pregnancies. The available literature on the topic is presented herein: 1) Fox et al. [18] collected 297 twin women divided into four groups based on their pBMI (underweight, normal weight, overweight, and obese). They compared pregnancy outcomes for women whose weight gain per week equaled or exceeded the IOM recommendations to women whose weight gain per week was lower than IOM cut-off, in three pBMI-based subgroups (underweight patients were excluded). They found that weight gain was associated with the gestational age at delivery and birth weight of the larger and smaller twin. Specifically, their study showed that, in women with a normal pBMI, patients whose weight gain met or exceeded the IOM recommendations had significantly improved outcomes, such as increment in birthweight of the larger twin and a lower rate of PD before 32 weeks (3.4% vs. 11.5%). In women with an overweight pBMI, if the weight gain met or exceeded the IOM recommendations, they reported higher gestational age at delivery, larger birth weight, and less preterm birth. In pre-gestational obese women, no statistically significant differences were noted; 2) The same authors [17] retrospectively studied a cohort of 170 women restricted to twin pregnancies at 37 weeks or more. Their analysis valued pregnancy outcomes in three groups based on IOM recommendations defined as poor, normal, and excessive weight gain. The rate of newborns weighing more than 2500 g was 40%, 60.5% and 79.5% in the three groups, respectively. No differences in gestational hypertension, pre-eclampsia, gestational diabetes or neonatal intensive care unit admission across groups were observed; 3) Gonzalez-Quintero et al. [19], aimed to determine the validity of IOM recommendations for weight gain in twin pregnancies in terms of impact on perinatal outcomes comparing women with mean weight gain per week meeting or exceeding recommendations versus patients who did not meet the suggested weight gain. There was a significantly higher number of both infants weighing > 2500 g or > 1500 g for women gaining weight at or above guidelines. Of interest, women whose gain was below recommended guidelines were 50% more likely to deliver spontaneously at < 35 weeks.

#### What do our study adds compared to the available literature

Our study follows, in line with the available literature, the idea of assessing whether changes in weight gain during pregnancy in twin gestations, may have an impact on maternal and perinatal outcomes. Moreover, our study design shows several peculiarities, which differentiate it from the previous reports.

Specifically, 1) considering that IOM recommendations were already pBMI correlated, we simplified our analysis and divided our population considering if patients met, exceeded, or presented lower gain weight according to pBMI IOM cut-offs, without performing further stratification of the study groups. The rationale for it was to make the influence of weight gain on PD clearer and useful in clinical practice. Indeed, our analysis showed that a normal weight gain, was correlated with better perinatal and maternal outcomes; 2) Compared with the analysis by Fox et al. [17], our study population also included twins delivered preterm at 28 weeks or more. This was done in order not to lose the effect of prematurity on the analyzed outcomes, since prematurity is common in twin gestations and different outcomes such as preeclampsia and SGA are more frequent in women delivering preterm; 3) In line with Quintero et al., we found that a weight gain below the recommended guidelines was associated with higher rates of spontaneous PD. Moreover, we compared normal weight gain both with under and over gain weight and did not associate patients who met or exceeded IOM recommendations. Of interest, we hypothesized that, both lower and excessive weight gain were associated with worse outcomes; our results confirmed the idea that pregnant women whose weight gain was over recommendation, are at higher risk of hypertensive disorder and PD.

#### Strengths and limitations of the study

The novelty of our work was to evaluate if there is an effect of IOM guidelines on the development of spontaneous PD in a cohort of twins at both term and preterm.

Moreover, our study has some limitations, mainly related to its retrospective design and to the small sample size as well as on the fact that it is built on a database registry.

Another possible weakness is the potential for missing data. To minimize this, at our hospital, data is reported by the obstetrician directly after delivery and skilled personnel routinely reviews the information before entering it into the database thereby minimizing recall bias. Coding was done after assessing the medical and prenatal care records together with the routine hospital documents. In addition, since there were no data regarding weekly weight gain cut-offs for twin pregnancies in IOM recommendations, we decided to apply linearity to weekly gain cut-offs as performed within IOM recommendations for single pregnancies [25].

## Conclusions

Our findings suggest that normal weight gain, according to revised IOM recommendations, is associated with a reduction of spontaneous PD and, in a selected population, with better pregnancy course and better obstetrics outcomes. This information could be useful for early counseling in twin pregnancy.

## Abbreviations

IOM: Institute of medicine
LGA: Large for gestational age
pBMI: Pre-gestational body mass index
PD: Preterm delivery
SGA: Small for gestational age
